# GM‐CSF antibodies in artificial stone associated silicoproteinosis: A case report and literature review

**DOI:** 10.1002/rcr2.1021

**Published:** 2022-08-11

**Authors:** Shana N. S. Khan, Robert G. Stirling, Catriona A. Mclean, Prudence A. Russell, Ryan F. Hoy

**Affiliations:** ^1^ Department of Respiratory Medicine Alfred Health Melbourne Victoria Australia; ^2^ Department of Medicine Monash University Melbourne Victoria Australia; ^3^ Department of Anatomical Pathology Alfred Health Melbourne Victoria Australia; ^4^ TissuPath Melbourne Victoria Australia; ^5^ Department of Epidemiology and Preventative Medicine, School of Public Health and Preventative Medicine Monash University Melbourne Victoria Australia

**Keywords:** occupational disease, pulmonary alveolar proteinosis, silica, silicoproteinosis, stone benchtop

## Abstract

Pulmonary alveolar proteinosis (PAP) is a rare lung disease where there is accumulation of surfactant in the alveoli. It can be classified based on the underlying aetiology into three categories: primary, secondary and congenital. Autoantibodies to granulocyte‐macrophage colony‐stimulating factor (GM‐CSF‐Ab) are a key diagnostic feature of autoimmune PAP. High intensity occupational exposure and inhalation of toxic particles such as silica can cause a form of secondary PAP called acute silicoproteinosis. We describe a 26‐year‐old stone benchtop fabricator with silicoproteinosis following daily exposure to high levels of silica who had elevated serum GM‐CSF‐Ab. We discuss the role of GM‐CSF‐Ab in cases of PAP with occupational inhalational exposure and the challenges in its interpretation.

## Graphical abstract

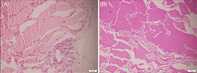

## INTRODUCTION

Pulmonary alveolar proteinosis (PAP) is a rare diffuse lung disease characterized by the accumulation of surfactant lipids and proteins in the alveoli and terminal airways due to defective clearance by alveolar macrophages.^1^, ^2^ Three main categories of PAP have been identified: primary, secondary and congenital.^3^

Autoimmune PAP (aPAP), a primary form of PAP that is the most common type in adults^3^ is driven by the presence of antibodies to granulocyte‐macrophage colony‐stimulating factor (GM‐CSF). Secondary PAP is less common and is associated with a range of clinical conditions that impair alveolar macrophage function and include malignancy, blood dyscrasias, immune deficiency syndromes, toxic dust inhalation as well as certain infections.^2^, ^3^, ^4^ Congenital PAP affects children and is due to genetic mutations affecting surfactant production.^2^, ^3^

We describe a case of silicoproteinosis in a young male who tested positive for serum GM‐CSF autoantibodies (GM‐CSF‐Ab) after working for nearly a decade as a stone benchtop fabricator.

## CASE REPORT

**FIGURE 1:**
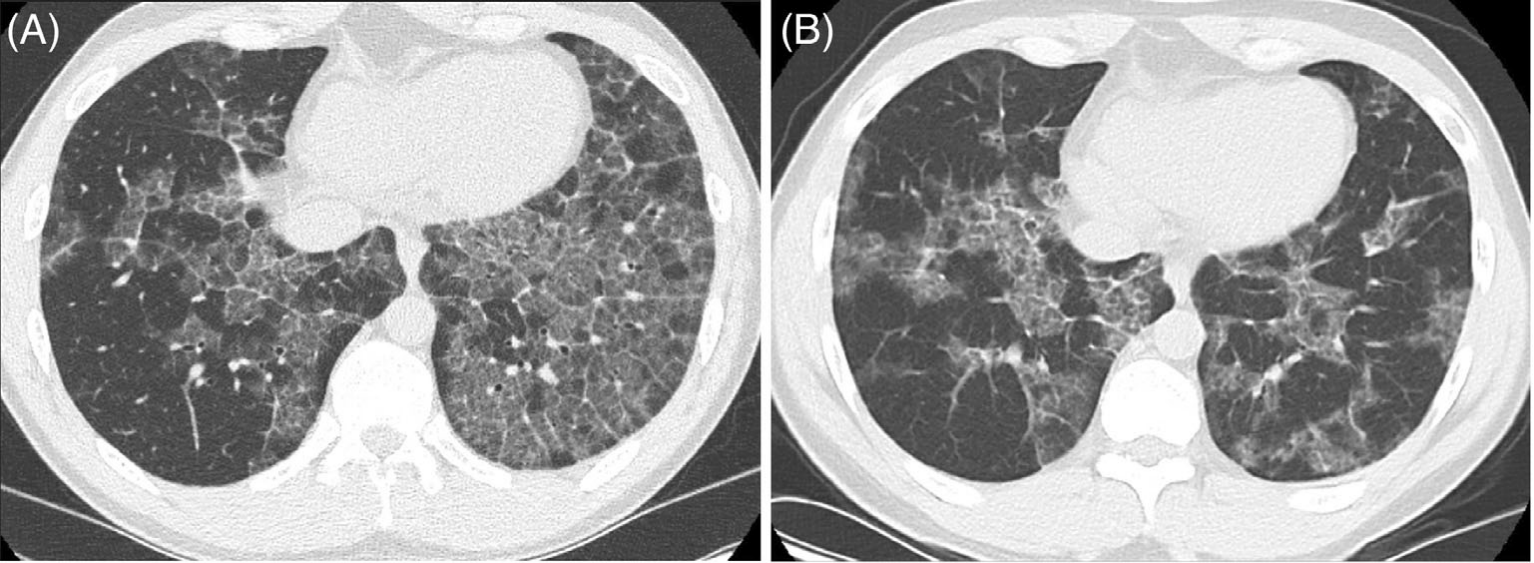
(A) Initial CT chest at diagnosis. (B) CT chest 2 years after eliminating silica dust exposure

A 26‐year‐old stone benchtop fabricator was referred with a 3‐year history of increasing shortness of breath on exertion, chest discomfort and bilateral crazy paving pattern on high‐resolution CT (HRCT) chest (Figure 1A). He had a significant history of atopy with lifelong asthma that was well controlled with low dose inhaled corticosteroid/long‐acting beta agonist therapy, allergic rhinitis, eczema and multiple food and flora allergies. He was an ex‐smoker with a 3‐pack year history and had been working in the stone benchtop fabrication industry for the past 8 years, dealing mainly with high silica content artificial stone.

He reported daily exposure to high levels of silica dust in his role of grinding and cutting artificial stone without water dust suppression. He did not use respiratory protection in the first 6 years and only started wearing a cartridge respirator intermittently in recent years.

Clinical examination was unremarkable with clear lung fields on auscultation, no evidence of cyanosis, clubbing or lymphadenopathy and normal oxygen saturations.

**FIGURE 2:**
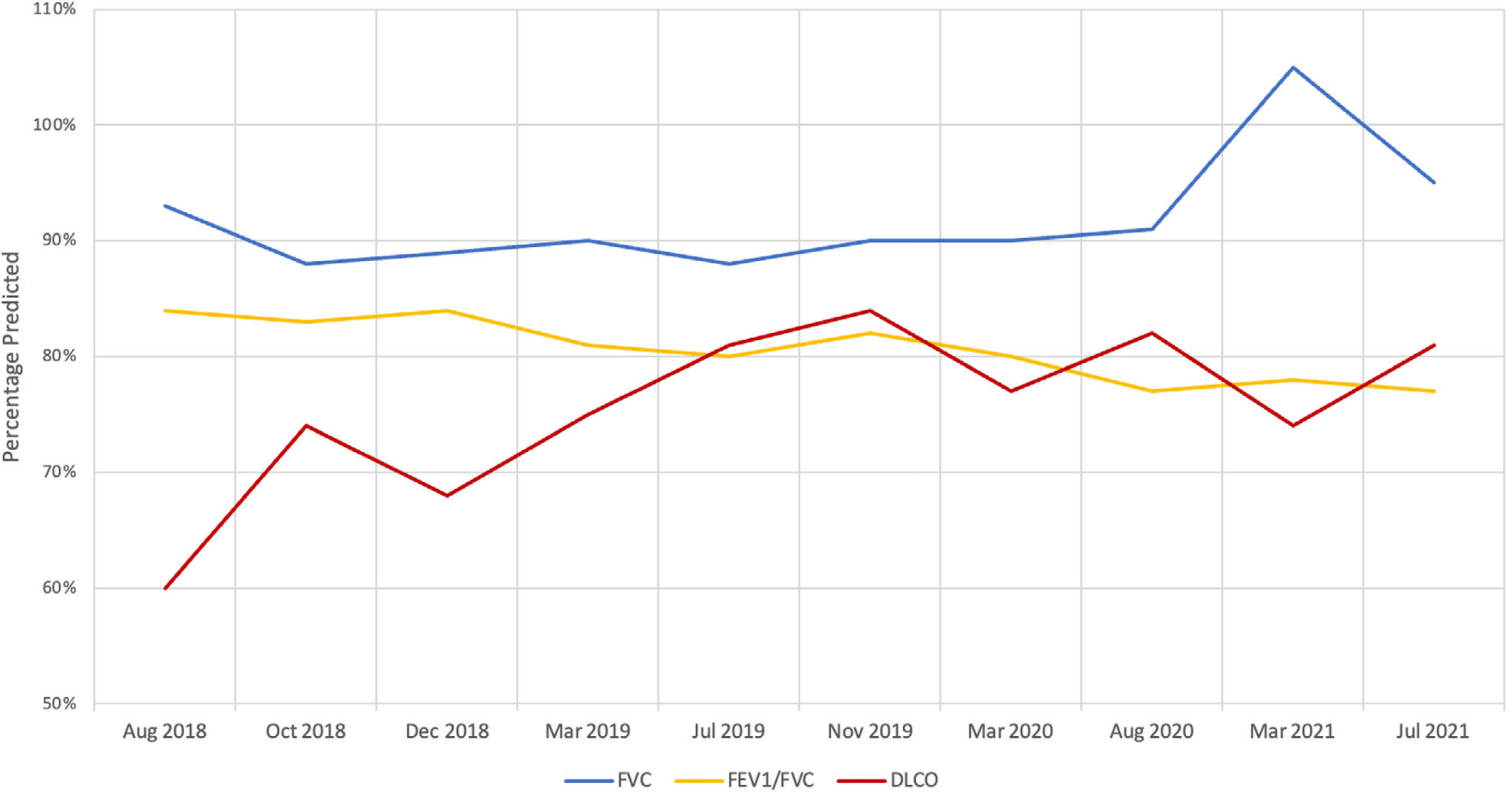
Patient's lung function test results over time. Last known occupational exposure to silica was in December 2018. FVC, forced vital capacity; FEV1/FVC, forced expiratory volume in 1 s/forced vital capacity; DLCO – single breath carbon monoxide diffusion capacity

**FIGURE 3:**
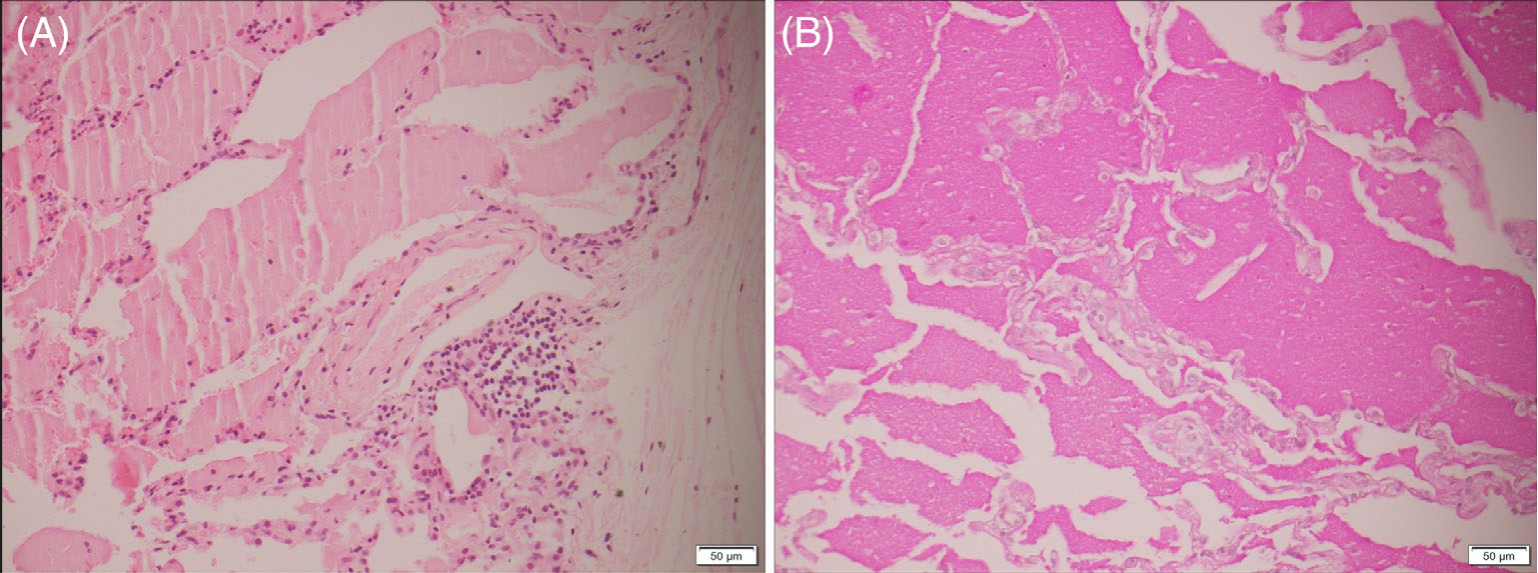
Alveolar proteinosis seen as alveolar spaces contain eosinophilic granular material (haematoxylin and eosin). (A) That is periodic‐acid Schiff (PAS) positive (B), in association with scant interstitial lymphocytes ×200 actual magnification

Initial lung function tests revealed a forced expiratory volume in 1 s (FEV_1_) of 92% predicted, forced vital capacity (FVC) of 93% predicted and single breath carbon monoxide diffusion (DLCO) of 22.9 ml/min/mmHg (60% predicted) (Figure 2). The bronchoalveolar lavage (BAL) fluid had a translucent and mucoid appearance. Microscopy identified granular proteinaceous material, pulmonary macrophages (84%) and acute (7%) and chronic (11%) inflammatory cells and was reported as benign. A subsequent open lung biopsy revealed alveoli filled with Periodic Acid‐Schiff stain positive proteinaceous material consistent with PAP (Figure 3A,B). Within the regions of PAP, areas of mild interstitial pneumonitis with foci of birefringent foreign material under polarized light were seen – highly suggestive of secondary PAP.

A second opinion was sought revealing elevated serum GM‐CSF‐Ab and low signal transducer and activator of transcription 5 (STAT5) phosphorylation levels. These findings were consistent with a diagnosis of aPAP but could not exclude secondary PAP.

In view of his inhalational history, lung function, radiological and histological findings, he was diagnosed with silicoproteinosis and discussed at a multi‐disciplinary meeting for consideration of treatment. However, as repeat lung function tests performed 4 months after baseline demonstrated stable results, the recommendation was to observe and monitor. The patient was advised to avoid further silica exposure and consequently left the stone benchtop industry.

A follow up HRCT chest 2 years following diagnosis demonstrated spontaneous reduction in crazy‐paving pattern particularly in the left lower lobe (Figure 1B). Lung function tests remained unchanged with reduced DLCO (Figure 2). He reported persistent, but not worsening, functional limitation with difficulty walking uphill and beyond two flights of stairs. Given the radiological improvement and stable physiological lung function, further intervention was not indicated, and he continues to undergo clinical, physiological and radiological surveillance.

## DISCUSSION

Secondary PAP is rare, accounting for 5%–10% of all cases of reported PAP.^5^, ^6^ It is predominantly associated with haematological disorders but has also been found to be associated with malignancy, chronic inflammatory disease and toxic inhalation.^2^, ^3^, ^4^, ^5^ Of all the inhalable toxic agents, silica was found to be the most common inhaled particle in workers who developed secondary PAP.^7^ This form of PAP following exposure to high concentrations of respirable crystalline silica is called acute silicoproteinosis.^2^, ^8^, ^9^ It was first recognized in the 1930s and named due to its histological resemblance to PAP.^8^

Secondary PAP is linked to a relative deficiency of GM‐CSF leading to either reduction in the number or function of alveolar macrophages, or both.^2^, ^3^, ^10^ Inhalation of high levels of toxic particles such as silica impair alveolar macrophage function through direct toxicity.^11^, ^12^ Acute exposure to extremely high levels of silica has also been found to lead to excess production of surfactant and proteinaceous exudate from type II alveolar epithelial cells and may also contribute to the development of acute silicoproteinosis.^11^

GM‐CSF is a key cytokine and colony stimulating factor involved in pulmonary homeostasis and host defence. It is produced by multiple cell types including alveolar epithelial cells, macrophages and lymphocytes.^2^, ^10^ As a colony stimulating factor it maintains adequate production and differentiation of granulocytes and macrophages via phosphorylation of STAT5 and subsequent activation of multiple intracellular signalling pathways.^5^ GM‐CSF also has additional effects on immunity through enhancing neutrophil bactericidal function and macrophage function through increased antigen presentation and phagocytosis.^10^ It has additional roles in host defence by promoting leukocyte migration via the production of numerous pro‐inflammatory cytokines.^2^, ^10^

In relation to PAP, GM‐CSF promotes the differentiation of alveolar macrophages in the human lung which serve to clear pulmonary surfactant and debris from the alveoli.^10^ Reduced numbers and function of alveolar macrophages from low GM‐CSF results in surfactant accumulation within alveoli.^2^ Low levels of GM‐CSF lead to reduced levels of PU.1, a transcription factor in alveolar macrophages. Reduction of PU.1 levels affect macrophage function by impairing catabolism of surfactant by alveolar macrophages causing surfactant accumulation.^2^, ^13^ These lowered levels of GM‐CSF and the build‐up of surfactant result in impaired host defences through loss of GM‐CSF mediated immunomodulation, reduced numbers of pulmonary granulocytes and macrophages, impaired leukocyte function, and physical effects of surfactant filled alveoli limited leukocyte migration and alveolar clearance.^8^, ^10^

GM‐CSF‐Ab neutralize the effects of GM‐CSF by interfering with the differentiation and maturation of alveolar macrophages resulting in the development of PAP.^2^ While high GM‐CSF‐CSF‐Ab levels of >5.0 μg/ml^6^, ^13^ have a sensitivity and specificity of 100% for aPAP,^6^, ^14^ the presence of GM‐CSF‐Ab itself is not pathognomonic for aPAP. In fact, low levels of GM‐CSF‐Ab may be present in healthy individuals, those with other interstitial lung disease and those with lung cancer.^2^, ^5^, ^6^

The presence of GM‐CSF‐Ab may be seen in some cases of secondary PAP. Borie et al. found that GM‐CSF‐Ab were detected in a patient with PAP secondary to indium inhalation.^2^ This suggests that toxic inhalation may be related to the development of antibodies to GM‐CSF.^2^ Certainly, silica exposure has been associated with autoimmune conditions such as rheumatoid arthritis, scleroderma and systemic lupus erythematosus (SLE).^9^, ^15^, ^16^ Studies have found that silica‐exposed individuals had high levels of circulating antibodies (IgA, IgG), anti‐nuclear antibodies (ANA) and anti‐neutrophil cytoplasmic antibodies (ANCA).^9^, ^15^, ^17^ While the mechanisms linking silica exposure and the development of autoimmune conditions remains unknown, it has been postulated that the strong immune response in the lung to respirable crystalline silica can induce an autoimmune reaction.^14^, ^15^, ^17^

GM‐CSF‐Ab levels also do not appear to correlate with disease duration, disease severity score, pulmonary function (FEV_1_, FVC, DLCO) or serum biomarkers (LDH, CEA, SP‐A, SP‐D)^13^ of PAP. Inoue et al. reported that GM‐CSF‐Ab concentrations were less than 35 μg/ml in most study patients but even in the top 13% of individuals with GM‐CSF‐Ab levels greater than 35 μg/ml, no correlations were observed in any of the parameters.^13^

Current knowledge about silicoproteinosis is limited and based on case reports and series. Unlike aPAP where spontaneous improvement is possible in 17%–25% of cases,^6^ the prognosis for secondary PAP is unpredictable and strongly influenced by the underlying disease.^2^ The progressive nature of silicoproteinosis makes its prognosis unfavourable, with most reports documenting mortality within months of diagnosis.^18^, ^19^, ^20^ A case report published by Ordonez‐Gomez et al. described a patient who clinically improved after removal from further silica exposure and who remained radiologically and physiologically stable 4 years after diagnosis.^21^ Similarly, Hemvimon et al. reported a patient who improved clinically after discontinuing silica exposure.^22^ As far as we are aware, our patient is the third reported case with documented clinical improvement following elimination of further silica exposure.

Treatment options for silicoproteinosis are limited and generally supportive.^2^, ^8^, ^9^ Aside from discontinuing further exposure, whole lung lavage (WLL) has been reported as a promising intervention as it clears the accumulated surfactant from the airspaces.^3^, ^9^ Several case reports suggest some utility of WLL in silicoproteinosis, but the clinical benefit remains unclear due to the lack of large cohort or randomized trial data.^3^, ^23^, ^24^

In the case of our patient, we found no other known aetiological reason for PAP than occupational exposure to silica dust. The relevance of elevated GM‐CSF‐Ab in the setting of silica exposure is not clear. It may be related to a degree of autoimmunity or alternatively explained by their presence in a proportion of the population, or a non‐specific or cross‐reactive antibody result. The diagnosis of silicoproteinosis, a secondary form of PAP, is supported by the histological findings on lung biopsy, and the improvement in radiological features and stable lung function following avoidance of silica dust exposure.

## AUTHOR CONTRIBUTION

*Manuscript conceptualisation*: Ryan F. Hoy and Robert G. Stirling. *Manuscript preparation*: Shana N. S. Khan. *Data Collection*: Shana N. S. Khan. *Manuscript review & final approval*: Shana N. S. Khan, Robert G. Stirling, Ryan F. Hoy and Prudence A. Russell.

## CONFLICT OF INTEREST

None declared.

## ETHICS STATEMENT

The authors declare that appropriate written informed consent was obtained for the publication of this manuscript and accompanying images.

## Data Availability

The data that support the findings of this study are available from the corresponding author upon reasonable request.
